# Developing a clinical decision tool to support paramedics when assessing and managing children with minor head injury

**DOI:** 10.1186/s12873-025-01362-1

**Published:** 2025-10-09

**Authors:** Alyesha Proctor, Mark D. Lyttle, Sarah Voss, Jonathan Benger

**Affiliations:** 1 Faculty of Health and Applied Sciences, University of the West of England, Glenside Campus (1H30), Blackberry Hill, Stapleton, Bristol, BS16 1DD UK; 2 Emergency Department, Perth Children’s Hospital, Perth, Australia

**Keywords:** Pre-hospital, Paramedic, Children, Head-injury, Decision tools, Delphi

## Abstract

**Background:**

Head-injured children are commonly transported to the Emergency Department (ED) by ambulance. However, most of those conveyed are deemed non-serious and are discharged at triage. Hospital clinicians use clinical decision tools to support their assessment of head-injured children; however, this is generally to determine whether a computed tomography (CT) scan is indicated. Currently, there is no pre-hospital clinical decision tool designed to support paramedics when assessing and managing head-injured children at scene. The aim of this study was to determine consensus amongst experts and stakeholders to inform the development of a new tool to support paramedics in safely assessing and managing children with minor head injury.

**Methods:**

A consultation process using a modified online Delphi technique comprising two rounds and a consensus meeting was completed between September 2023 and January 2024. A 5-point Likert scale was used to assess consensus, set a-priori at 67%. Free text survey responses arising from the Delphi were studied and concepts were developed. Data were analysed anonymously, and feedback was given after each round.

**Results:**

An expert stakeholder group comprising 36 participants took part in Round One, and 34 participants in Round Two of the online Delphi. The participants included parents/grandparents/caregivers, paramedics, primary care clinicians, ED doctors, ED nurses and Paediatricians. Consensus was reached in 36 statements following Rounds One and Two. The remaining eight statements were discussed at a consensus meeting, which was attended by 12 stakeholders. Seven of the eight statements reached agreement.

**Conclusion:**

This Delphi study has established consensus amongst a group of experts and stakeholders on the content and format of a pre-hospital paediatric head injury clinical decision tool, designed for use by paramedics: **PATCH** (Pre-hospital Assessment Tool for Children with Head injury). Future research should include an evaluation of the acceptability and usability of PATCH by paramedics.

**Clinical trial number:**

Not applicable.

**Supplementary Information:**

The online version contains supplementary material available at 10.1186/s12873-025-01362-1.

## Background

Globally, head injury in children is a common reason for Emergency Medical (ambulance) Services (EMS) activation. In the UK, there are approximately 800,000 attendances to Emergency Departments (ED) each year for childhood head injury, a third of which are transported by ambulance [1]. 90% of these are minor, requiring no specific intervention, and three-quarters of those conveyed by ambulance in the UK are deemed non-serious and discharged at triage [2]. Avoidable ambulance transport to the ED incurs costs and can result in poor patient and parent experience [3].

Several hospital-based tools exist to support clinical decision-making when a child presents to the ED with a head injury [4]. These are generally designed to identify patients who may require a computed tomography (CT), or neurosurgical intervention. NICE [1] head injury guidance is of limited utility to paramedics for reasons including: a lack of validation in pre-hospital care; reference to patient observation over time which is not always possible; challenges in the assessment of headache and amnesia in younger children; and limited research evidence on which to base recommendations relating to ambulance non-conveyance [1].

Currently, there is no pre-hospital clinical decision tool to support paramedics in determining the need for conveyance of children with head injury [5]. Such a tool has the potential to reduce ED attendances, conserve resources and provide better patient and parent experience, whilst ensuring safe and effective clinical care [6].

This gap is recognised in existing national guidance [1] and has never been more important in the context of rising ambulance service demand, long response times and policy initiatives to avoid ED conveyance when it is safe and appropriate to do so [7].

## Research question

What is the consensus view of a group of experts and stakeholders regarding the ideal content and format of a tool designed to support paramedics in safely assessing and managing children with head injury?

## Methods

An online modified Delphi study consisting of two rounds and a consensus meeting was completed between September 2023 and January 2024. The consensus meeting took place online via Microsoft Teams on the 03/01/2024. Round One and Round Two ran for one month each, with a maximum of three reminders sent in each round, if participants had not responded. The first reminder was sent two weeks after launch, then one week before closing and finally 24 h before closing. The online survey was delivered using Qualtrics [8], which is a secure, web-based software platform. Data were stored securely on a university encrypted computer within the lead researcher’s organisation. An online meeting took place in January 2024 to discuss any statements that did not reach consensus.

### Population

The eligibility criteria are displayed in Table 1 below.

**Table 1:** Eligibility criteria:

Inclusion Criteria	Exclusion Criteria
Parents/Grandparents/Care-givers with experience of their child suffering a head injury that required seeking medical help.	Parents/Grandparents/Care-givers with no experience of their child suffering a head injury.
Paramedics working for a UK ambulance service with experience of attending to children who have sustained a head injury.	Paramedics working in an alternative setting to the ambulance service.
General Practitioners (Doctors), Advanced Clinical Practitioners (nurses or paramedics by background) working in primary/urgent care in or out of hours.	GPs/ACPs who do not see children in day-to-day practice.
Emergency Department Doctors who regularly see head injured children.	Emergency Department Doctors who work in an adult only facility.
Emergency Department Nurses who regularly see head injured children	Emergency Department Nurses who work in an adult only facility.
Paediatricians with a specialist interest/expert knowledge in paediatric head injury	Paediatricians without a specialist interest in paediatric head injury.
Worldwide	

### Recruitment and sample size

The target sample was at least 30 participants, notionally composed of five laypeople, five paramedics, five GPs/ACPs, five ED doctors, five ED nurses and five paediatricians with specialist knowledge in paediatric head injury. The decision to include a heterogeneous sample was purposeful, since different stakeholders have varying opinions on the assessment and management of head-injured children, which enriches results. The inclusion of patients *and* healthcare professionals combined within Delphi methodology is often encouraged [9]. A recent scoping review found that only a quarter of published Delphis use patients [10], despite clear evidence that effectively engaging patients in research about their care or their child’s care is essential to improve health outcomes [11].

Recruitment was consecutive based on expressions of interest and eligibility, until the desired sample size was reached [12]. Laypeople (parents/grandparents/caregivers) were recruited directly from an established Parent Advisory Group during a Patient and Public Involvement meeting. These individuals had experience of their child/grandchild suffering a head injury and needing to contact the ambulance service/ED. All parents in the group were informed about what would be involved in the Delphi process during the meeting. Paramedics, doctors, nurses, GPs and ACPs were invited to contact the researcher using their NHS email address if they were interested in participating, via social media and professional connections.

All participants were emailed following Round Two of the Delphi to seek interest in the final consensus meeting. The first two participants from each stakeholder group who expressed their interest in taking part were recruited.

### Enrolment and consent

The 38 stakeholders who expressed an interest in participating were sent an email containing a participation information sheet, information on the Delphi process, consent form, and a link to the survey. Consent could be confirmed by return of consent form, or within the online Delphi questionnaire. Participants were made aware that they had the right to withdraw from the study at any time until their data was analysed. Every participant has provided their informed consent to participate.

### Data collection

The statements in this Delphi were developed following the results of a systematic review [5] and interview study [13]. The review aimed to identify which elements of hospital-based clinical decision support tools for head-injured children could be used in pre-hospital care. The interview study considered the factors influencing paramedic conveyance decisions when attending children with minor head injury. The Delphi did not include statements on signs of a seizure, signs of skull fracture, drug or alcohol use or complex wound management. These clinical criteria were judged to be fundamental to include in the tool following the systematic literature review and qualitative interviews with paramedics, and therefore a consensus approach using Delphi methodology was not required.

Statements were provided with Likert responses, and ability to make free text comments. This allowed participants to expand where they wished, supplying the researcher with written information for feedback and development of subsequent rounds. Following analysis of Round One, participants were sent individual feedback on their answers compared with the rest of the group, which was entirely anonymised. The anonymous report of the group result did not include free text comments where participants had justified their answers, as in the absence of the ability to have a dialogue on any free text, this could influence participants unduly. Participants were asked to compare their individual response with the group response, particularly on questions that had not reached consensus, before completing the next round. In Round Two, participants were sent a document explaining the rationale for modifying questions or including new questions.

Once statements that had reached consensus were removed, Round Two of the Delphi survey was distributed. Those who did not complete Round One were ineligible to complete subsequent rounds, and no new invitations were sent. Participants were sent individual feedback and an anonymous group report following Round Two of the Delphi survey. As only eight statements had not reached consensus it was decided to hold a consensus workshop to discuss the remaining statements, rather than a third online round.

### Data analysis

An a-priori criterion of 67% agreement consistent with other studies [14] was used to assess whether statements were acceptable or not acceptable for inclusion in the clinical decision tool. Data were analysed anonymously, and responses were grouped into three categories of ‘Agree’ (strongly agree/agree), ‘neither agree nor disagree’, and disagree (strongly disagree/disagree).

For statements on which consensus was reached, means and medians were calculated from the five-point Likert scale results, to represent overall agreement and ranking. The highest percentage (whether that be agree or disagree) was calculated for each question to identify whether consensus was achieved. Free text comments arising from the Delphi process were used to modify statements for subsequent rounds and in discussion during the consensus meeting. These comments also shaped the final development of the tool. A description of the concepts arising from the free text comments in the Delphi study are shown in Table 3, while illustrative quotes from both rounds are reproduced in Table S6 (supplementary material).

## Results

An expert group made up of 36 participants (out of a possible 38 originally recruited) took part in Round One of the online Delphi, with 34 taking part in Round Two. The participants included parents/grandparents/caregivers with experience of their child suffering a head injury (*n* = 5), paramedics (*n* = 9), General Practitioners/Advanced Clinical Practitioners in primary care (*n* = 6) (*n* = 5 Round Two), ED doctors (*n* = 5) (*n* = 4 Round Two), ED nurses (*n* = 8), Paediatricians with expert knowledge in paediatric head injury (*n* = 3).

Participant demographics are shown in Table 2.

**Table 2:** Participant demographics

Participant ID	Demographics				
	Expert group	Ethnicity	Age	Gender	Location
PAR 1	Patient	White British	25–35	Male	UK Southwest
PAR 2	Patient	White British	Over 55	Female	UK Southwest
PAR 3	Patient	White other	25–35	Female	UK Southwest
PAR 4	Patient	White British	25–35	Female	UK Southwest
PAR 5	Patient	White British	25–35	Female	UK Southwest
PAR 6	Paramedic	White British	25–35	Male	UK Northeast
PAR 7	Paramedic	White British	25–35	Female	UK Northwest
PAR 8	Paramedic	White British	25–35	Female	UK Midlands
PAR 9	Paramedic	U/K	25–35	Male	UK Midlands
PAR 10	Paramedic	White British	18–24	Male	UK Southwest
PAR 11	Paramedic	White British	36–45	Female	UK Southeast
PAR 12	Paramedic	U/K	25–35	Male	UK Midlands
PAR 13	Paramedic	White British	25–35	Male	Wales
PAR 14	Paramedic	White British	36–45	Male	UK midlands
PAR 15	GP	White British	46–55	Male	UK Southwest
PAR 16	ACP	White British	25–35	Female	UK Southwest
PAR 17	GP	White British	36–45	Male	UK Southwest
PAR 18	ACP	White British	25–35	Male	UK Southwest
PAR 19	ACP	White British	36–45	Female	UK Southwest
PAR 20	GP	White British	36–45	Female	UK Southwest
PAR 21	ED Doctor	White British	25–35	Female	UK Southeast
PAR 22	ED Doctor	White British	25–35	Male	UK Northeast
PAR 23	ED Doctor	British Asian	36–45	Male	UK Southeast
PAR 24	ED Doctor	Pakistani	36–45	Male	UK Southeast
PAR 25	ED Doctor	White British	36–45	Male	UK Midlands
PAR 26	ED Nurse	White British	36–45	Female	UK Southwest
PAR 27	ED Nurse	White	36–45	Female	UK Southwest
PAR 28	ED Nurse	White British	46–55	Male	UK Southwest
PAR 29	ED Nurse	Hispanic	25–35	Female	UK Southwest
PAR 30	ED Nurse	White British	36–45	Female	UK Southeast
PAR 31	ED Nurse	White British	36–45	Female	UK Southwest
PAR 32	ED Nurse	White British	36–45	Female	UK Southwest
PAR 33	ED Nurse	White British	36–45	Female	UK Southwest
PAR 34	Paediatrician	White	36–45	Male	UK Southeast
PAR 35	Paediatrician	White other	45–55	Male	Outside of the UK (Australia)
PAR 36	Paediatrician	White British	45–55	Male	UK Midlands

**Fig. 1:**
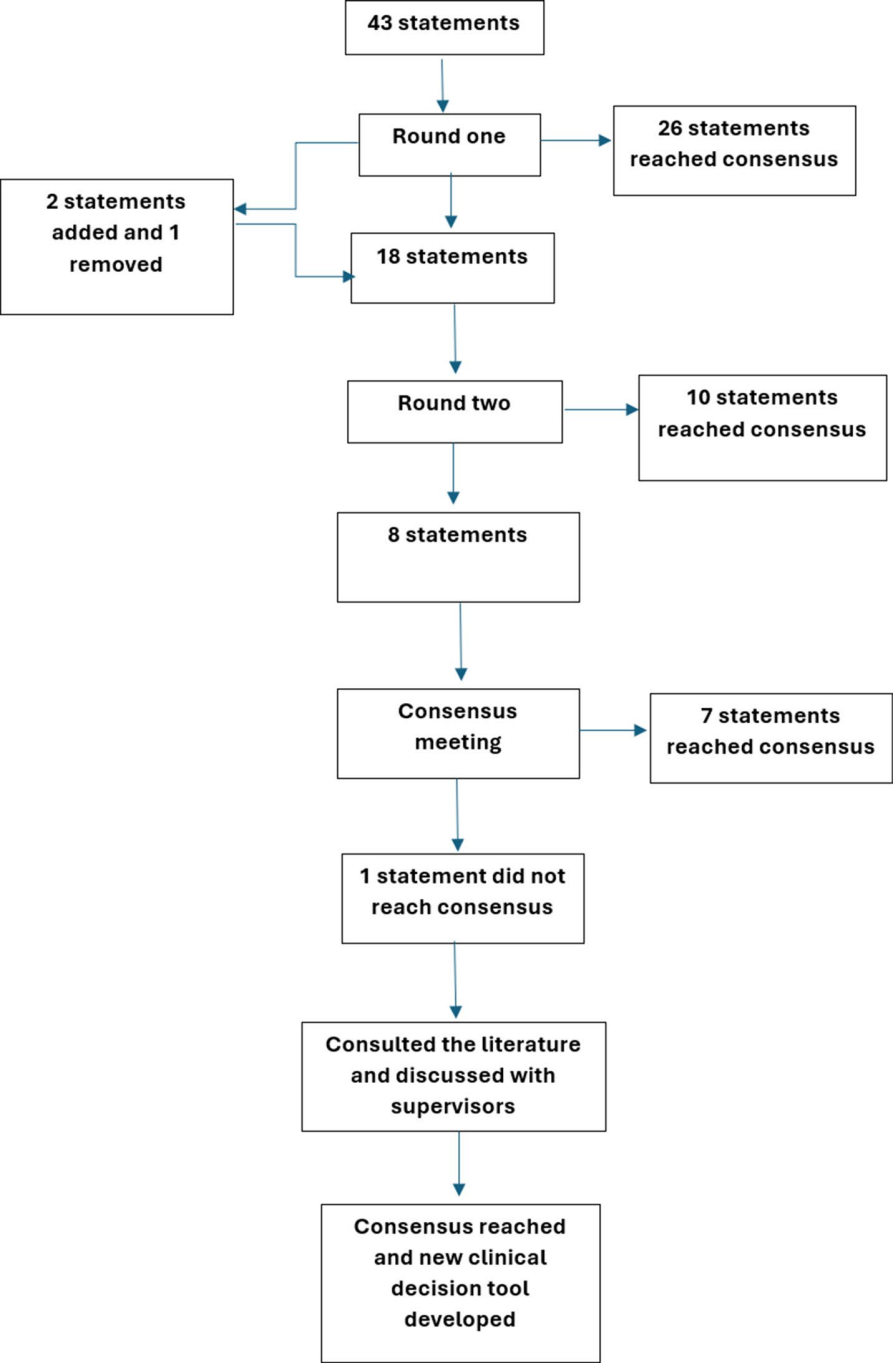
Flow of statements through the study

Consensus was reached in 26/43 statements in Round One and these were removed from further rounds. Two additional statements were included in Round Two and one removed: ‘A child with a head injury should be conveyed to the Emergency Department if they are reported to have lost consciousness for at least five seconds’ as this did not reach consensus, but participants did reach consensus on ‘a child should be conveyed to the ED with any amount of time of loss of consciousness’ and therefore this superseded it. Consensus was reached on 10/18 statements following Round Two. This left eight statements to be discussed in the consensus meeting of which seven reached the threshold for consensus. Figure 1 demonstrates the flow of statements through the study.

The detail of responses for statements in Round One is presented in Table S1 (supplemental material). Statements that were modified (and additional statements) following Round One in response to free text comments are shown in Table S2. The breakdown of responses for statements in Round Two is presented in Table S3. The eight statements that did not reach consensus following both rounds and were discussed in the consensus meeting are presented in Table S4. The outcome, justification and points of discussion for the eight statements considered in the consensus meeting are summarised in Table S5.

Free text comments arising from the Delphi process were used to modify statements for subsequent rounds and in discussion during the consensus meeting. These comments also shaped the final development of the tool. A description of the concepts arising from included criteria are shown in Table 3, while further detail on the concept and illustrative quotes from both rounds are reproduced in Table S6 (supplementary material).

**Table 3:** Concepts arising from free text comments

Clinical criterion	Concept
Vomiting	‘Not in isolation’ ‘It’s all about timing’ ‘The more vomits, the more concern’
Mechanism of injury	‘Warrants observation, too risky’ ‘Subjective nature’ ‘Safeguarding’
Bleeding disorder/clotting	‘Seek advice from a senior clinician’ ‘Sole antiplatelet not a problem’ ‘Consideration of CT’
Bruise/haematoma	‘If it’s boggy’ ‘Younger age greater concern’ ‘Size matters’
Age	‘Non accidental injury is vital here’ ‘If unsure, just convey’
Loss of consciousness	‘Shocked or unconscious’ ‘Not in isolation’ ‘Managing expectations’
Neurological deficit/vertigo/amnesia/mental status	‘Challenging in younger children’ ‘Reduced GCS is significant’
Headache	‘Severity is key’ ‘Associating symptoms’ ‘Parental conveyance’
Parental concern and capability	‘Parents know best’ ‘Safety netting’
Time since injury	‘Time alone not an important factor’ ‘Longer wait equals reassured’
Additional factors	‘Postcode lottery’ ‘Inclusivity for all’ ‘Newly qualified’ ‘Staying is not necessary’ ‘Constructive feedback’
Format	‘Paper is still needed’ ‘Flow chart or App’

### PATCH (Pre-hospital Assessment Tool for Children with Head injury)

**Fig. 2:**
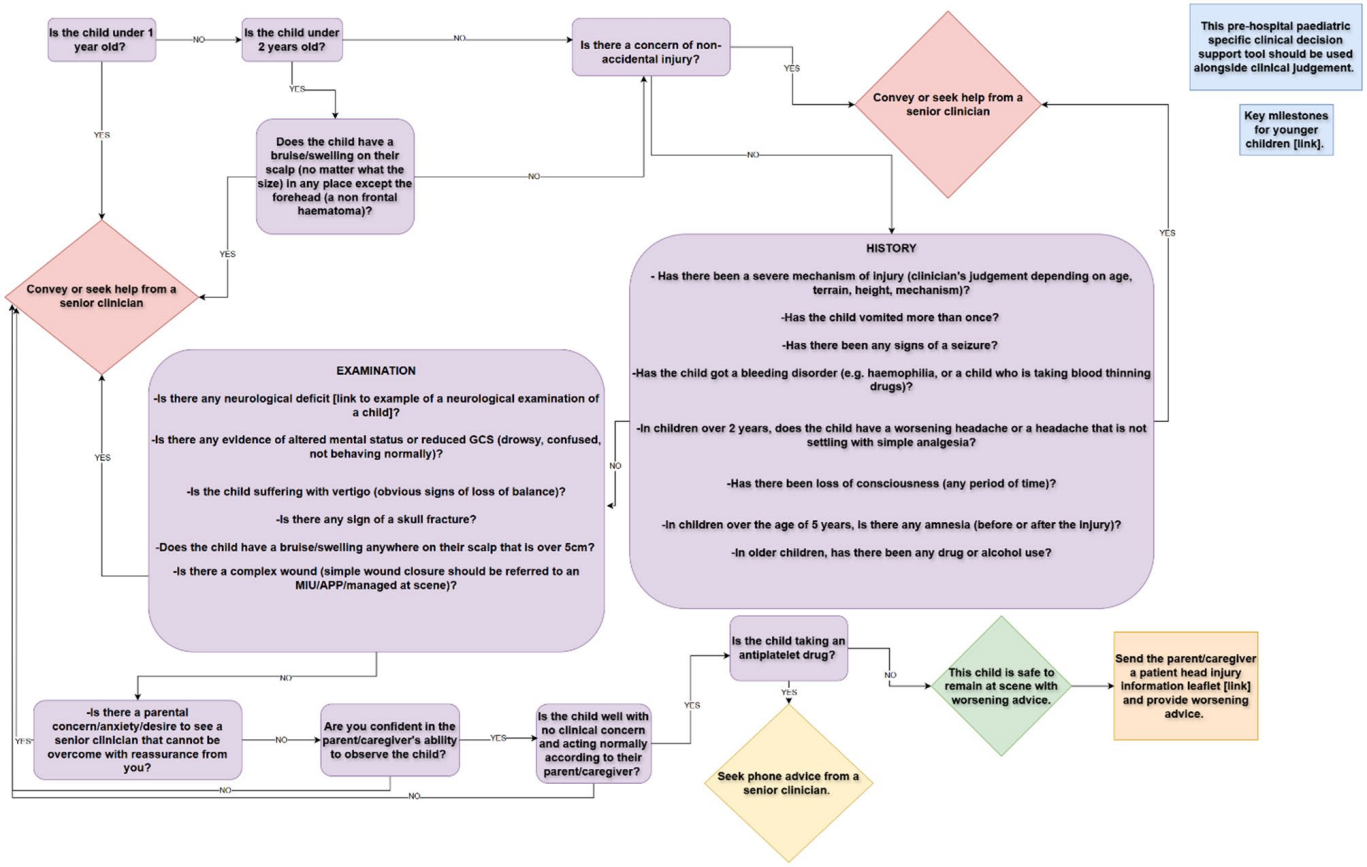
PATCH (Pre-hospital Assessment Tool for Children with Head injury) flowchart

Following completion of the Delphi process, the clinical criteria for the tool were finalised and version 1.0 of PATCH (Pre-hospital Assessment Tool for Children with Head injury) was drafted. Figure 2 shows the flow chart, and a progressive web application version is also available: PATCH.

## Discussion

Through this Delphi study, consensus was achieved on 43 statements that have informed the development of a Pre-hospital Assessment Tool for Children with Head injury (PATCH), for use by paramedics. Delphi studies are a well-established method for seeking consensus [15] and are particularly useful for developing clinical decision tools [16].

### Vomiting

Participants were first asked a series of questions related to a head-injured child vomiting. The number of vomits deemed to indicate observation or CT scanning varies across in-hospital tools [5]. Therefore, consensus was required to decide the number of vomits that should be included in a pre-hospital tool, or whether it should be a clinical criterion at all. Children are more likely to vomit following a head injury, regardless of their intracranial pressure [17]. In this Delphi study, consensus was agreed in Round One that if a child vomits more than once then this warrants conveyance to hospital. In Round Two it was agreed that if the child vomits only once, but has no further vomits, then they are safe to remain at home with “safety-netting” advice. NICE [1] advises that if a child has three episodes of discreet vomiting, then this warrants observation for at least four hours, with any further vomiting requiring a CT scan. This begs the question of whether a child who has vomited twice should be taken to the hospital, as this does not warrant observation according to NICE [1]. However, PATCH is designed to be highly sensitive for clinically significant traumatic brain injury. Therefore, the final decision was made to include ‘has the child vomited more than once’ within the tool.

### Mechanism of injury

What constitutes a ‘severe’ mechanism of injury varies considerably within hospital-based clinical decision tools for children with head injury [5]. Additionally, evidence regarding whether a severe mechanism of injury as a sole criterion is a significant factor for clinically significant traumatic brain injury is mixed. Mechanism of injury alone is not predictive of an abnormal brain CT [18], but it is still a common reason to arrange this investigation [19]. This suggests that clinicians are unnerved by mechanism of injury, even if the child appears well with no other “red flag” symptoms. Consensus about the inclusion of a severe mechanism of injury in the tool as a sole criterion for conveyance was reached in Round Two, in favour of inclusion.

Severe mechanism of injury as a reason for conveyance is reflected in other studies with older adults who have fallen and sustained a head injury [20]. Determining what is meant by a ‘severe’ mechanism of injury is complex. This is particularly the case for children because they are different ages and different heights. For example, NICE [1] suggest a fall of over 3 m is a dangerous mechanism, however as argued by experts in this Delphi, a fall of 1 m could be severe if a child is only 1 year old and landed directly on their head. Even the most validated and accepted in-hospital clinical decision tools for children with head injury (PECARN, CHALICE and CATCH) have very varying descriptions of ‘severe’ mechanism of injury. At the consensus workshop it was agreed that paramedics should use their own clinical judgement on whether the mechanism was severe.

### Bleeding disorder/clotting

Participants were asked one question about anticoagulants and bleeding disorders and one about antiplatelet drugs. The evidence for the significance of taking anticoagulants or antiplatelets when a child hits their head is very limited [17]. None of the three most widely validated tools (PECARN, CATCH and CHALICE) include ‘anticoagulant use/clotting disorder’ as a clinical predictor [4]. However, NICE [1] advocate a CT head scan within one hour if a child has a bleeding/clotting disorder along with one other clinical criterion. Babl and colleagues [21] identified gaps in the current most validated in-hospital head injury tools (PECARN, CHALICE and CATCH) which included bleeding disorder/anticoagulation and antiplatelet use. They developed a new guideline which advised clinicians to consider structured observation over immediate CT scan, and to consult senior clinicians including the team managing the anticoagulation therapy/blood disorder. This suggests that conveyance is required for these patients.

In this Delphi, participants agreed in Round One that anticoagulants should be included in the tool; however, this was mainly based on adult evidence. Consensus was not achieved on whether antiplatelets should be included and this was discussed in the consensus meeting. Participants suggested that this would be a rare scenario, and that Aspirin monotherapy is less significant according to adult guidelines and is contraindicated in under 16-year-olds [22]. It was decided that a question on antiplatelets should be included in the tool, however the paramedic would discuss this situation with a senior clinician and not necessarily convey the child if there were no other clinically concerning symptoms.

### Bruise/haematoma/swelling

Participants were asked questions related to the size and location of bruises/swellings/haematomas in children who have sustained a head injury. The risk of skull fracture and associated intracranial haemorrhage is correlated with both scalp haematoma size and location [23]. This is particularly if the swelling is over the temporal or parietal lobe and ‘large’ [23]. Delphi participants agreed that if there is a bruise/swelling/haematoma that is larger than 5 cm in any age child they should be referred to the ED. This is again more risk averse since hospital-based tools like CHALICE include this as a criterion, but for under 1-year olds only [24].

Participants concluded that the consideration of bruise/swelling/haematoma should be for all ages and not just under 2-year-olds. Scalp haematomas in infants are more indicative of intracranial injury than older children and this is particularly the case if they are boggy or non-frontal [25]. Despite this, consensus amongst the participants was not reached for ‘a child under the age of 2 years who has a bruise/swelling on their scalp (no matter what the size) in any place accept the forehead (a non-frontal haematoma) should be conveyed to ED’. Clinicians in the consensus meeting felt that this would result in too many unnecessary attendances to the ED, however parents felt they should be conveyed.

PECARN is the most widely validated tool used in hospital for head injured children and is extremely sensitive with very few false negatives and excellent negative predictive values. Therefore, if PECARN is followed, it is highly unlikely a clinically important brain injury will be missed [26]. It includes a non-frontal haematoma as a criterion for under 2-year-olds [27]. Due to the evidence and the fact this criterion is in PECARN, the decision to include non-frontal haematoma in the tool was made, for under 2-year-olds only. The term ‘boggy’ was not included in the tool specifically, since this was felt to be encompassed by ‘signs of a skull fracture’.

### Age

PECARN is the only hospital-based tool that differentiates between age groups [27]. Consensus was reached amongst participants in this study that questions should be specific to a child’s age, as for example it would be difficult to assess a child under the age of 5 with amnesia. The PATCH tool begins by asking if the child is under 1 year of age. Evidence suggests that children under the age of 1 can be asymptomatic with intracranial injury, skull fractures can occur despite minor trauma and abusive head trauma occurs more frequently [25]. The conveyance policies relating to children amongst UK ambulance services vary considerably. Most policies are based on guidance from the RCPCH in 2009 that recommended that all children under the age of 2 should be conveyed, however some trusts have moved away from this [28]. Ambulance service policy still stipulates that an under 1 year old with an injury should be conveyed to the ED [28]. This is mainly due to safeguarding concerns, which is supported by the literature; sadly, abusive head trauma is the leading cause of fatal head injuries in infants [29]. Consensus in this Delphi study was to not include under 1-year olds in the final tool.

### Loss of consciousness

Hospital based tools vary on the duration of loss of consciousness that potentially could indicate a clinically important brain injury [5]. Therefore, participants in this Delphi study were presented with statements related to what duration of loss of consciousness warrants conveyance. Consensus was reached in Round One (97% of participants) that children who have lost consciousness for over five minutes should be conveyed to the ED. Consensus was also reached in Round One that if a child has lost consciousness for any amount of time that they should be conveyed to the ED. Reasons for this included parental expectation and the need for observation. NICE [1] head injury guidelines suggest a period of four hours observation if the child has a loss of consciousness for over five minutes as an isolated finding. However, the PREDICT guideline for children with mild to moderate head injury suggests that a loss of consciousness for over five seconds in under 2-year-olds and any amount of time for over 2-year-olds should be followed by four hours of observation [21]. This is in line with PECARN [27].

The evidence is therefore unclear as to what duration is more likely to be suggestive of significant brain injury, and when a child should be observed. It was agreed that PATCH should specify that a child with loss of consciousness for any amount of time should be conveyed to the ED. Children with minor head trauma who suffer an isolated loss of consciousness are at very low risk for clinically important brain injury [30], however they still warrant a period of observation which cannot be undertaken by a paramedic in routine practice.

### Neurological deficit/vertigo/amnesia

Altered mental status (drowsy, confused or not behaving normally) achieved 100% consensus to be included in PATCH during Round One. This is supported by evidence; altered mental status following a head injury, including confusion, disorientation, and not behaving normally, can be a firm indicator of intracranial injury [31].

The tool also asks separately about amnesia and vertigo. The evidence for the inclusion of vertigo in the hospital-based tools is limited, and it is one of the most infrequently used criteria for identifying clinically significant intracranial injury [1, 5]. Originally, in Round One, the inclusion of vertigo as a stand-alone clinical criterion resulted in a 61% agreement. When asked again in Round Two, 71% of participants agreed that it should be included. 85% of participants agreed that amnesia should be included in the tool. Interestingly, PECARN does not include amnesia as a clinical criterion despite there being a link between duration of amnesia and severity of a head injury [32].

### Headache

Headache is a common symptom in head-injured children [33]; however, consensus was reached in this study that not every child with a headache following a head injury needs conveyance to hospital. This is supported by evidence that suggests a headache in a child following a head injury is more likely to indicate significant brain injury if it is prolonged, severe or associated with other clinical criteria [34]. The results of this Delphi indicated that a head-injured child with a prolonged or severe headache that does not settle with simple analgesia should be conveyed to the ED. Participants reported in the free text that headache is difficult to assess in younger children, which is reflected in the evidence [21].

### Time since injury

Paramedics consistently report that they feel more confident to discharge a head-injured child at scene if a longer period has elapsed since injury [13]. Of the children that have a fatal outcome from a head injury, almost all of them present with clinical features that make it clear they need an urgent admission soon after injury (e.g. reduced conscious level) [1]. Four hours is the usual time allocated to observe a head injured child in the ED. This is based on very little evidence and is a consensus view and established practice from experts with specialist knowledge in the field [35]. Therefore, the Delphi study asked participants whether children should be taken to the ED if their head injury occurred less than four hours ago. The same was also asked for under two hours.

Consensus was reached that time since injury should not be the sole reason to convey a head-injured child to hospital if there are no other significant clinical criteria. Many children are discharged at triage when they arrive at the ED, and this could be 30 min to hours after the injury [36].

### Parental concern and capability

Patient acting normal as per parent/caregiver is an uncommon criterion in hospital-based tools, with only two (PECARN and the HIDATq) including it as an important factor [5]. Contradictory to this, 92% of participants in this Delphi study reported that it should be included within the pre-hospital tool as a clinical criterion, reaching consensus when first asked. Consensus was also reached that the newly developed tool should not exclude children with a developmental delay, unlike many in-hospital tools such as CATCH [37]. In these cases, it is even more important to involve the child’s parents in their care.

94% of participants agreed that confidence in the parent’s or caregiver’s capability to observe should be included as a clinical criterion in the tool. Following the head injury (if the child is not taken to the ED), they need to be observed and monitored by a responsible parent/caregiver for 48 h [25]. If this is not possible then the child should be conveyed to a place of safety.

### Format

PATCH has been designed to exclude “red flags”, so even one clinical criterion present will result in either conveyance or seeking advice from a senior clinician. This is to ensure the tool is highly sensitive, and therefore able to “rule out” clinically significant brain injury if there are no features of concern, allowing the child to be left safely at scene with “safety netting” advice. Support for both a flow chart and electronic version was identified in the Delphi.

## Limitations

The participants in the study included only one person from outside the UK, despite being open to recruitment globally. Therefore, the findings may not apply to other settings, such as low resource countries. PATCH may be particularly useful in these environments, since children in low-resource countries face inadequate medical infrastructure and delayed assessment and management. There were only three paediatricians recruited to the study, whereas other stakeholder groups such as ED nurses had eight participants. An equal number of all stakeholders may have achieved greater balance and proved preferable, though there were equal numbers from each stakeholder group at the consensus meeting. The sample was predominantly from a White British ethnicity, and those from a black, Asian or minority ethnic group were underrepresented at only 5/36. Two participants did not disclose their ethnicity.

## Conclusions

This Delphi study established consensus amongst a group of experts and stakeholders on the clinical criteria that should be included in a novel pre-hospital paediatric head injury clinical decision tool, designed for use by paramedics: PATCH (Pre-hospital Assessment Tool for Children with Head injury). Future research should include initial evaluation of the acceptability and usability of PATCH by paramedics. Following this, further evaluation is required to assess the validity and test characteristics of the final tool and examine safety, without changing clinical practice. If validity and safety are confirmed, further research will need to determine whether the introduction of PATCH for paramedics reduces the rate of ambulance conveyance to the ED for children with head injury, while avoiding adverse outcomes and improving patient and parent experience.

## Supplementary Information

Below is the link to the electronic supplementary material.

Supplementary Material 1

## Data Availability

Data is provided within the manuscript or supplementary information files.

## Declarations

### Patients and the public

Patients and public have been involved throughout this research. Both a Young Persons Advisory Group (children aged 9 to 17 years from Bristol and surrounding area) and Parent Advisory Group (parents and grandparents from the Southwest of England) have supported this study and confirmed its importance to children, parents, and the wider health and care system. Five members of the PAG were involved in doing the Delphi from a parent/patient point of view.

### Transparency statement

This manuscript is an honest, accurate and transparent account of the study being reported. No important aspects of the study have been omitted and any discrepancies from the study as originally planned have been explained.
