# Supplementary material for: Developing a clinical decision tool to support paramedics when assessing and managing children with minor head injury
Source: BMC Emerg Med. 2025 Oct 9;25:203. doi: 10.1186/s12873-025-01362-1 (PMC12512835; doi:10.1186/s12873-025-01362-1)
Supplement: Supplementary file 1 — Supplementary Material 1 [file 12873_2025_1362_MOESM1_ESM.docx]

**Supplementary Material**

**Table S1. Round One statements and level of consensus**

| *Round One statements* | *Highest percentage consensus (disagree, neutral, agree) to be included in the tool.* | *Outcome* | *Consensus reached?* |
| --- | --- | --- | --- |
| A child who vomits once should be conveyed to the ED (no other clinically worrying symptoms). | 58% | Disagree | NO |
| A child who vomits more than once should be conveyed to the ED. | 67% | Agree | YES |
| A child should be conveyed to ED if they have had at least 3 distinct episodes of vomiting. | 92% | Agree | YES |
| A child who has sustained a head injury but shows no concerning clinical features should still be conveyed to the Emergency Department if the “mechanism of injury” (description of how the head injury occurred) is judged to be severe. | 56% | Agree | NO |
| A fall of one metre should be classed as a severe mechanism of injury (for any age child). | 39% | Agree | NO |
| A fall of over two metres is a severe mechanism of injury. | 75% | Agree | YES |
| A fall of more than three metres is a severe mechanism of injury. | 100% | Agree | YES |
| A child with a bleeding disorder (e.g. haemophilia, or a child who is taking blood-thinning drugs) and who has sustained a head injury should be conveyed to the Emergency Department, even if they have no concerning clinical features. | 92% | Agree | YES |
| A child who is taking antiplatelet drugs (e.g. aspirin) and who has sustained a head injury should be conveyed to the Emergency Department, even if they have no concerning clinical features. | 53% | Agree | NO |
| A child who has a bruise/swelling on their forehead (a frontal haematoma) following a head injury, but with no concerning clinical features can remain at home with advice to recontact the ambulance service immediately if they deteriorate. | 84% | Agree | YES |
| A child under the age of 2 who has a bruise/swelling on their scalp (no matter what the size) in any place except the forehead (a non-frontal haematoma) should be conveyed to the Emergency Department. | 39% | Agree | NO |
| A scalp bruise/swelling (haematoma) should only be considered in the decision to convey a child to the Emergency Department in those under 2 years of age (i.e. birth to 24 months old). | 53% | Disagree | NO |
| A child under the age of 1 year (i.e. birth to 12 months old) who has sustained a head injury should be conveyed to the Emergency Department, even if there are no safeguarding concerns or concerning clinical features. | 47% | Agree | NO |
| A child under the age of 2 years (i.e. birth to 24 months old) who has sustained a head injury should be conveyed to the Emergency Department, even if there are no safeguarding concerns or concerning clinical features. | 75% | Disagree | YES |
| A child under the age of 2 years (i.e. birth to 24 months old) who has sustained a head injury should be discussed with a senior clinician (paediatrician or GP), even if there are no safeguarding or concerning clinical features if they are not conveyed to the Emergency Department. | 50% | Disagree | NO |
| Vertigo (dizziness or a loss of balance) should be included in a pre-hospital clinical decision tool to support paramedics in managing head-injured children. | 64% | Agree | NO |
| A child who has sustained a head injury and has lost consciousness for any amount of time should be conveyed to the Emergency Department. | 78% | Agree | YES |
| A child with a head injury should be conveyed to the Emergency Department if they are reported to have lost consciousness for at least five seconds. | 61% | Agree | NO |
| A child with a head injury should be conveyed to the Emergency Department if they are reported to have lost consciousness for at least five minutes. | 97% | Agree | YES |
| If a child has a headache following a head injury, they should be conveyed to the Emergency Department (no other clinically concerning features). | 47% | Disagree | NO |
| If a child has a headache that is getting worse following a head injury, they should be conveyed to the Emergency Department. | 83% | Agree | YES |
| If a child has a headache that is not settling with simple pain relief following a head injury, they should be conveyed to the Emergency Department | 64% | Agree | NO |
| Amnesia (loss of memory) before or after the injury, should be included in a pre-hospital clinical decision tool to support paramedics in managing head-injured children. | 69% | Agree | YES |
| Amnesia (loss of memory) should be included in a pre-hospital clinical decision tool to support paramedics in managing head-injured children, but for older children only (over the age of 5), as it would be difficult to assess amnesia in younger children. | 56% | Agree | NO |
| Altered mental status (drowsy, confused or not behaving normally) should be included in a pre-hospital clinical decision tool to support paramedics in managing head-injured children. | 100% | Agree | YES |
| ‘Patient well with no clinical concern and acting normal according to a parent or caregiver’ should be included in a pre-hospital clinical decision tool to support paramedics in managing head-injured children. | 92% | Agree | YES |
| Confidence in the parent’s or caregiver’s capability to observe should be included in a pre-hospital clinical decision tool to support paramedics in managing head-injured children. | 94% | Agree | YES |
| Time since injury should be included in a pre-hospital clinical decision tool to support paramedics in managing head-injured children. | 86% | Agree | YES |
| If the time since injury is less than four hours, head-injured children should be conveyed to the Emergency Department. | 67% | Disagree | YES |
| If the time since injury is less than two hours, head-injured children should be conveyed to the Emergency Department, even if there are no concerning clinical features or red flags. | 64% | Disagree | NO |
| Distance from the hospital and whether the parent or caregiver has transport readily available should be included in a pre-hospital clinical decision tool to support paramedics in managing head-injured children. | 47% | Agree | NO |
| A pre-hospital clinical decision tool to support paramedics in managing head-injured children should not be used on children with a developmental delay. | 36% | Disagree | NO |
| A pre-hospital clinical decision tool to support paramedics in managing head-injured children should be available to all levels of paramedic, including newly qualified paramedics. | 91% | Agree | YES |
| Paramedics should be able to remain on scene with a head-injured child for an hour after examination and assessment to observe them and avoid unnecessary conveyance to the Emergency Department, which may take more time. | 50% | Disagree | NO |
| Feedback given by hospital doctors and nurses to paramedics about what happens to a child once they have been brought to the hospital would help to reduce conveyance of head injured children. | 83% | Agree | YES |
| Most children with head injury can be observed safely at home, rather than being conveyed to an Emergency Department. | 89% | Agree | YES |
| A pre-hospital clinical decision tool to support paramedics in managing head-injured children should be available in multiple languages. | 81% | Agree | YES |
| A pre-hospital clinical decision tool to support paramedics in managing head-injured children should have different questions depending on the age of the child (be age specific). | 89% | Agree | YES |
| A pre-hospital clinical decision tool to support paramedics in managing head-injured children should include key milestones to remind paramedics what a child is usually able to do at certain ages. | 83% | Agree | YES |
| A pre-hospital clinical decision tool to support paramedics in managing head-injured children should be presented as a flow chart. | 75% | Agree | YES |
| A pre-hospital clinical decision tool to support paramedics in managing head-injured children should be presented in an electronic format. | 97% | Agree | YES |
| A pre-hospital clinical decision tool to support paramedics in managing head-injured children should be incorporated into the routine electronic patient report form (where paramedics input patient data). | 83% | Agree | YES |
| An advice leaflet that can be electronically emailed or texted to the parent or caregiver, if a child is not conveyed to the Emergency Department, would be beneficial. | 97% | Agree | YES |

**Table S2. Modifications to statements following Round One**

| **Statement** | **Modification** | **Justification** |
| --- | --- | --- |
| A child who vomits once should be conveyed to ED. | A child who vomits once should be conveyed to ED (no other clinically concerning symptoms). | Modified to reflect free text comments saying that the answer depends on whether this is associated with other clinically concerning symptoms. |
| A fall of one metre should be classed as a severe mechanism of injury. | A fall of one metre should be classed as a severe mechanism of injury (for any age child). | Modified to reflect free text comments saying that it would be dependent on the child’s age. |
| A child who has a bruise/swelling on their scalp in any place except their forehead (a non-frontal haematoma) should be conveyed to the ED. | A child under the age of 2 who has a bruise/swelling on their scalp (no matter what the size) in any place except their forehead (a non-frontal haematoma) should be conveyed to the ED. | Modified to reflect free text comments saying that it is dependent on age and the size of the haematoma. |
|  | A child under the age of 2 who has a bruise/swelling anywhere on their scalp that is over 5cm should be conveyed to the ED, even if there are no other clinically concerning features. | Additional statement following free text comments on the size of the haematoma. This is also present in multiple hospital-based clinical decision tools. |
| If a child has a headache following a head injury, they should be conveyed to the ED. | If a child has a headache following a head injury, they should be conveyed to the ED (no other clinically concerning symptoms). | Modified wording in response to free text comments saying it is dependent on whether there are other clinically concerning symptoms. |
| Amnesia (loss of memory) should be included in a pre-hospital clinical decision tool to support paramedics in managing head injured children, but for older children only (over the age of 5). | Amnesia (loss of memory) should be included in a pre-hospital clinical decision tool to support paramedics in managing head injured children, but for older children only (over the age of 5), as it would be difficult to assess amnesia in a younger child. | Modified wording following free text comments asking why age 5 is the set age. |
|  | Parental concern should be included in a pre-hospital clinical decision tool to support paramedics in managing head-injured children. | Additional question for Round Two. This should have been in Round One following PPI input. |
| If the time since injury is less than two hours, head-injured children should be conveyed to the ED. | If the time since injury is less than two hours, head-injured children should be conveyed to the ED, even if there are no concerning clinical features/red flags | Modified wording following free text comments saying that it is dependent on whether there are any red flags present. |
| Paramedics should be able to remain on scene with a head injured child for an hour after examination and assessment and observe them. | Paramedics should be able to remain on scene with a head injured child for an hour after examination and assessment and observe them, and avoid unnecessary conveyance to the ED, which may take more time. | Additional wording in response to free text comments about paramedics not having time. |

**Table S3. Round Two statements and level of consensus**

| ***Round Two Statements*** | ***Highest percentage consensus (disagree, neutral, agree)*** | ***Outcome*** | ***Consensus reached?*** |
| --- | --- | --- | --- |
| A child who vomits once should be conveyed to the ED (no other clinically concerning symptoms) | 91% | Disagree | YES |
| A child who has sustained a head injury but shows no concerning clinical features should still be conveyed to hospital if the mechanism of injury is judged to be severe. | 74% | Agree | YES |
| A fall of one metre should be classed as a severe mechanism of injury (for any age child). | 44% | Disagree | NO- discuss in consensus meeting |
| A child who is taking an antiplatelet drug (e.g.) Aspirin and who has sustained a head injury should be conveyed to the ED, even if they have no clinically concerning symptoms. | 62% | Agree | NO- discuss in consensus meeting |
| A child under the age of 2 who has a bruise/swelling on their scalp (no matter what the size) in any place except the forehead (a non-frontal haematoma) should be conveyed to the ED. | 44% | Agree | NO- discuss in consensus meeting |
| A child under the age of 2 who has a bruise/swelling anywhere on their scalp that is over 5cm should be conveyed to the ED even if there are no other concerning symptoms. | 76% | Agree | YES |
| A scalp bruise/swelling (haematoma) should only be considered in the decision to convey a child to the ED in those under 2 years of age. | 76% | Disagree | YES |
| A child under the age of 1 year who has sustained a head injury should be conveyed to the ED, even if there are no safeguarding concerns or clinically concerning symptoms. | 50% | Agree/disagree | NO- discuss in consensus meeting |
| A child under the age of 2 years who has sustained a head injury should be discussed with a senior clinician (paediatrician or GP) even if there are no safeguarding concerns or clinically concerning symptoms. | 56% | Disagree | NO- discuss in consensus meeting |
| Vertigo should be included in a pre-hospital clinical decision tool to support paramedics in managing head-injured children. | 71% | Agree | YES |
| If a child has a headache following a head injury, they should be conveyed to the ED (no other clinically concerning features). | 74% | Disagree | YES |
| If a child has a headache that is not settling with simple pain relief following a head injury, they should be conveyed to the ED. | 82% | Agree | YES |
| Amnesia should be included in a pre-hospital clinical decision support tool to support paramedics in managing head-injured children, but for older children only (over 5 years) as it would be difficult to assess amnesia in a younger child. | 85% | Agree | YES |
| Parental concern should be included in a pe-hospital clinical decision tool to support paramedics in managing head-injured children. | 74% | Agree | YES |
| If the time since injury is less than 2 hours, head-injured children should be conveyed to the ED, even if there are no clinically concerning symptoms. | 85% | Disagree | YES |
| Distance from the hospital and whether the parent or caregiver has transport readily available should be included in a pre-hospital clinical decision tool to support paramedics in managing head injured children. | 59% | Agree | NO- discuss in consensus meeting |
| A pre-hospital clinical decision tool to support paramedics in managing head-injured children should not be used on children with a developmental delay. | 53% | Disagree | NO- discuss in consensus meeting |
| Paramedics should be able to wait on scene with a head-injured child for an hour after examination and assessment to observe them and avoid unnecessary conveyance to the ED, which may take more time. | 47% | Disagree | NO- discuss in consensus meeting |

**Table S4. Statements not reaching consensus after round 2, and therefore discussed at the consensus meeting**

| A fall of one metre should be classed as a severe mechanism of injury (for any age child).  **(44% disagree)** |
| --- |
| A child who is taking an antiplatelet drug (e.g.) Aspirin and who has sustained a head injury should be conveyed to the ED, even if they have no clinically concerning symptoms.  **(62% agree)** |
| A child under the age of 2 who has a bruise/swelling on their scalp (no matter what the size) in any place except the forehead (a non-frontal haematoma) should be conveyed to the ED.  **(44% agree)** |
| A child under the age of 1 year who has sustained a head injury should be conveyed to the ED, even if there are no safeguarding concerns or clinically concerning symptoms.  **(50% disagree/agree)** |
| A child under the age of 2 years who has sustained a head injury should be discussed with a senior clinician (paediatrician or GP) even if there are no safeguarding concerns or clinically concerning symptoms.  **(56% disagree)** |
| Distance from the hospital and whether the parent or caregiver has transport readily available should be included in a pre-hospital clinical decision tool to support paramedics in managing head injured children.  **(59% agree)** |
| A pre-hospital clinical decision tool to support paramedics in managing head-injured children should not be used on children with a developmental delay.  **(53% disagree)** |
| Paramedics should be able to wait on scene with a head-injured child for an hour after examination and assessment to observe them and avoid unnecessary conveyance to the ED, which may take more time.  **(47% disagree)** |

**Table S5. Consensus meeting outcomes**

| **Statement** | **Consensus outcome** | **Comments** |
| --- | --- | --- |
| A fall from one metre should be classed as a severe mechanism of injury for any age child. | Exclude | Just have ‘severe mechanism of injury’ as a criterion and the severity is dependent on the clinician’s judgement, taking into consideration the height, terrain, age etc. Participants agreed that the tool should not include specifics because the list would be too long. They felt that a paramedic would be able to differentiate between what constitutes a severe mechanism of injury and what does not. |
| A child who is taking antiplatelet drugs, for example Aspirin and who has sustained a head injury should be conveyed even if there are no clinically concerning symptoms. | Include but not to send to ED. Speak to a paediatrician for advice. | Rare scenario as children not often taking these medicines.  If there are no other clinically concerning symptoms, but the child is taking Clopidogrel then phone call to paediatrics if possible (if not GP). The may observe them for a longer period of time. Aspirin monotherapy would be less significant according to adult guidelines, and Aspirin is contraindicated in under 16-year-olds. |
| A child under the age of 2 years who has a bruise/swelling on their scalp (no matter what the size) in any place accept the forehead (a non-frontal haematoma) should be conveyed to ED. | The consensus was to exclude on the point of view of the clinicians; however, parents disagreed. Evidence does suggest that a non-frontal haematoma is more indicative of intracranial haemorrhage. This is particularly the case for infants. PECARN includes a non-frontal haematoma as a criterion for under 2-year-olds. CHALICE only includes size (identified in WP1 systematic review) | Senior clinicians have not found this to be a useful predictor in clinical practice.  They feel that size of the haematoma is more important than the location |
| A child under the age of 1 who has sustained a head injury should be conveyed, even if there are no clinically concerning symptoms. | Include | Paramedics reported a lack of equipment to fully assess a child of this age. They also reported a lack of confidence with this age group.  Current policy is to convey under 2-year-olds, but the consensus was very much that this is not needed.  If it has been a significant wait time for the ambulance and the child is completely fine, then consider phoning a GP or paediatrician before discharge, rather than automatic conveyance to ED purely because of age.  Safeguarding concern for this age group, although this is more for under 6-month-olds/non-mobile. There will be a question asking about safeguarding concern anyway.  There was unanimous consensus that under 3-month-olds will always need to be reviewed by a paediatrician. |
| A child under the age of 2 years should be discussed with a senior clinician even if there are no clinically concerning symptoms or safeguarding concerns. | Exclude (although this would mean changing ambulance policy) | Parents felt that this would be reassuring.  1–2-year-olds are a group who hit their heads all of the time because they are learning to walk/run, so this could be a lot of calls to GPs and paediatricians.  If the child has no red flags and no safeguarding concerns, then being 20 months old does not put them at any more risk than a 3-year-old of a significant brain injury. |
| Distance from the hospital and whether the parent has transport readily available should be included as a criterion in the tool. | Exclude | It was felt that this would result in a post code lottery.  Not to use as a specific criterion within this tool, however the paramedic would look at the social factors as they would for any call and take this into consideration. |
| The tool should not be used on children with developmental delay. | Exclude (the tool can be used on any child no matter the developmental delay). | The tool should still be able to be used for children with developmental delay with the help of carers and parents to identify what is normal for them. Not to be included as a criterion or an exclusion criterion. |
| Paramedics should be able to wait at scene to observe for an hour. | Exclude | It was thought that if the paramedics felt the patient needed further observation that could not be done by the parent themselves then they should be conveyed.  There is likely to have been a wait for the ambulance to arrive anyway, and with the time it takes to examine the child and complete paperwork, this would be a good period of observation. |

**Table S6. Concepts arising from the free text comments in both rounds of the Delphi process**

| **Criterion** | **Concept** | **Illustrative Quote** |
| --- | --- | --- |
| Vomiting | ‘Not in isolation’:  Participants regularly reported that it was dependent on other factors and that vomiting in isolation is not indicative of clinically significant traumatic brain injury. This question was modified for Round Two to state that there are no other clinically concerning symptoms. | *“I would factor in other symptoms, no evidence to support one isolated vomit”*  *[PAR 36]* |
| Vomiting | ‘It’s all about timing’:  Participants reported that it depends on the timing of the vomiting, and whether the vomiting was associated with crying or a viral illness. | *“Specially if the vomiting episode did not occur immediately after the head injury, as children can often vomit once straight after the injury as a result of intense crying/upset”.*  *[PAR 29]* |
| Vomiting | ‘The more vomits, the more concern’:  Most participants were not worried by one episode of vomiting following the head injury, especially if it was immediately after the injury and associated with crying. However, the more episodes of vomiting, the more participants felt this at least warranted observation in ED, particularly as more than three episodes is in the NICE guidance (NICE, 2023). | *“I think given the NICE guidance it would be difficult for a paramedic not to action attending ED after 3 vomits, although I personally disagree with the current evidence”.*  *[PAR 36]* |
| Mechanism of injury | ‘Warrants observation, too risky’:  Most participants commented that they feel patients who have sustained a head injury from a severe mechanism warrant observation. They reported that it would be too risky to leave a child at home with a significant mechanism of injury because they would struggle to defend that decision. Some participants felt that observation could be done at home with sensible parents, whereas others felt this should be in the ED. However, it was a common thought that what is deemed a significant mechanism is subjective and difficult to judge. | *“I’d struggle to comfortably discharge a child if there was any head injury with a high mechanism of injury. I believe a child would probably be just observed but I would be more comfortable if this was done in ED”.*  *“If a child is well and parents are sensible then not unreasonable to observe at home, this requires strong communication skills and worsening advice”.*  *[PAR 13]* |
| Mechanism of injury | ‘Safeguarding’:  Safeguarding was also a common concern reported by the participants. | *“Mechanism of injury and parental explanation would be key. Any concern of NAI must be considered”.*  *[PAR 17]* |
| Mechanism of injury | ‘Subjective nature’:  Hospital based tools specify different factors that warrant a ‘significant’ mechanism of injury. This includes what height constitutes as significant. Participants were asked specifically about what height constitutes a substantial mechanism of injury. They commonly wrote that this is dependent on the child’s age, what surface they landed on and how they landed. This demonstrates the complexity of deciding what is a high mechanism of injury, and the long list of factors to consider. | *“It would be dependent on the type of fall - ie rolling off a bed or bouncing off of a trampoline. They could be very different impacts, but from the same height.”*  *[PAR 1]* |
| Bleeding disorder/clotting | ‘Seek advice from a senior clinician’  It was felt that those on anticoagulants should be observed in the ED for consideration of a CT and those on sole antiplatelet would not require conveyance but potentially discussion with a senior clinician. | *“I would probably seek advice in this instance [antiplatelets], I don’t think I would convey the patient, but I am using adult guidelines as a thought process”.*  *[PAR 13]* |
| Bleeding disorder/clotting | ‘Sole antiplatelet not a problem’  Participants commonly reported a lack of evidence in this area for paediatrics. Most of them suggested that if the patient had a bleeding disorder or were taking anticoagulants that they would utilise adult evidence to make their decision. They felt that anticoagulants were more of a concern than antiplatelets (particularly sole antiplatelet use). They also reported this would be an unlikely scenario since Aspirin is contraindicated in under 16-year-olds. | *“No clinical need for conveyance if only single antiplatelet. There is no evidence of increased risk of intracranial bleeding”.*  *[PAR 12]* |
| Bleeding disorder/clotting | ‘Consideration of CT’  It was felt that those on anticoagulants should be observed in the ED for consideration of a CT and those on sole antiplatelet would not require conveyance but potentially discussion with a senior clinician. | *“A period of observation is beneficial for children on anticoagulants even if a CT is not required during admission.”*  *[PAR 22]* |
| Bruise/swelling/  haematoma | ‘If it’s boggy’  Participants did not seem to comment on the location being non-frontal as more of a concern. Participants commonly reported that they were more concerned about a swelling if it was boggy/ showing signs of a skull fracture. | *“Size and other characteristics (boggy/firm) define whether a bruise of swelling is clinically significant not just the presence alone.”*  *[PAR 22]* |
| Bruise/swelling/  haematoma | ‘Younger age greater concern’  They felt that this symptom was more important for younger children under the age of 2, particularly coming from a safeguarding point of view. | *“Dependent on age, if under 1 should be conveyed”.*  *[PAR 19]* |
| Bruise/swelling/  haematoma | ‘Size matters’  Participants commonly reported that they were more concerned if the size was over 5cm and this was particularly if the child was younger. | *“Safeguarding should be considered here. The size and extent is important”*  *[PAR 10]* |
| Age | ‘Non accidental injury is vital here’  The overriding concept when asked about whether under 1-year olds should be taken to the ED despite appearing very well following a head injury is the concern of a non-accidental injury. Most participants felt that under 1-year olds should be seen by a paediatrician because of this reason. It was felt that information sharing was vital in these circumstances. | *“I typically refer or convey all this age group (under 1s) to the ED, partly due to confidence and exposure, but particularly here I would be very concerned about non accidental injury”.*  *[PAR 13]* |
| Age | ‘If unsure, just convey’  It was also clear that if paramedics felt under confident that a child under 2 should be able to remain at scene then to just take them to the ED without contacting another clinician, such as a GP, as they do not see head injuries very often. | *“GPs have little experience in dealing with head injuries in children in day-to-day practice, if paramedics are in a position that they cannot soundly decide to leave a patient at home, then just bring them to the ED”.*  *[PAR 28]* |
| Loss of consciousness | ‘Shocked or unconscious’  There was a unanimous agreement that a loss of consciousness for over five minutes was a significant cause for concern, but if only for a few seconds this could be where the child was in shock, rather than true loss of consciousness. | *“A few seconds of ‘stunning’ rather than true LOC can be difficult to assess”.*  *[PAR 21]* |
| Loss of consciousness | ‘Not in isolation’  Similarly to vomiting, participants commonly reported that loss of consciousness is not indicative of significant intracranial injury as an isolated finding. | *“There is very little evidence to show association between LOC and the presence of traumatic brain injury on CT scan, it requires a second red flag to have CT imaging and can be considered to stay at home with safety netting”.*  *[PAR 34]* |
| Loss of consciousness | ‘Managing expectations’  It was acknowledged it would be difficult to manage parent expectations if there had been any period of loss of consciousness. | *“They would need conveyance to be observed, as I think this would benefit the parents and meet their expectations”.*  *[PAR 13]* |
| Neurological deficit/ vertigo/ amnesia/ mental status | ‘Challenging in younger children’  When considering assessment of vertigo and general neurological examination, participants reported that this would be more challenging to assess in younger pre-school age children. It was thought that vertigo would be a useful symptom to consider but for older children. Similar was reported for amnesia, where participants felt it was an important symptom but only for school age and above children. These questions were modified for Round Two to reflect the age comments. | *“Assessment of cognition in younger children can be difficult to ascertain especially asking them to recount memories about a certain event”.*  *[PAR 3]* |
| Neurological deficit/ vertigo/ amnesia/ mental status | “Reduced GCS is significant”  There were very little free text comments regarding reduced GCS/altered mental status as this was clearly deemed a vital symptom to include in the tool. | *“There are strong links between reduced GCS and presence of traumatic brain injury”.*  *[PAR 22]* |
| Headache | ‘Severity is key’  Most participants commented that a headache as an isolated symptom is not a cause for concern unless it is severe or prolonged. Several participants reported that it is important that the child has been given appropriate analgesia to see if that helps. | *“Depends on the headache severity and other presentations taken into account. Headache can be normal from a minor head injury and treated with pain relief”.*  *[PAR 7]* |
| Headache | ‘Associating symptoms’  Participants commonly reported that they would be more concerned if the headache was associated with other concerning symptoms such as visual changes and vertigo. | *“I would be more worried if it was associated with other symptoms such as visual changes, dizziness and vomiting”.*  *[PAR 31]* |
| Headache | ‘Parental conveyance’  Several participants wrote that parents could convey the child, rather than the ambulance crew if they needed to be observed in the ED. | *“I believe conveying in an ambulance can at times be stressful for children therefore if they’re suitable for self-conveyance then this is a great option.”*  *[PAR 12]* |
| Parent capability and concern: | ‘Parents know best’  It was clear from the free text comments that participants agreed that parental concern is very important and should be taken seriously. However, participants reported that this should be in relation to the child’s behaviour/gauge of patient’s baseline (as they know them best) rather than the parent being overly anxious even though their child is presenting normally. | *“Parents/carers views on how the child is presenting are important and should definitely be included in the tool”*  *[PAR 12]* |
| Parent capability and concern: | ‘Safety netting’  There were a limited number of free text comments regarding parent capability to observe but of those written, it is an important part of “safety netting” the child. | *“If you do not think a child will be safely and appropriately observed at home then they should be taken to the ED”.*  *[PAR 22]* |
| Time since the injury | ‘Time alone is not an important factor’  Participants commonly reported that time alone is not an important factor. Free comments suggest that time alone is not sufficient for a clinical decision, and it is only if other clinically important symptoms are present. | *“The rest of the assessment should be taken into consideration, time since injury is not sufficient for clinical decisions”.*  *[PAR 29]* |
| Time since the injury | ‘Longer wait equals reassured’  it was frequently reported that it would be more reassuring the longer the time since injury and the child remains well, and this was acknowledged. | *“I think if I see a child with a head injury that happened 5 minutes ago, my decision making is likely to be different versus an injury that occurred 2 hours ago, but I’d use red flags to make my decision”.*  *“If more than 4 hours ago and remain well then they can be reassured (generally)”*  *[PAR 25]* |
| Additional factors | ‘Postcode lottery’  It was generally reported that distance from the hospital should not be included in a pre-hospital tool because this could promote a postcode lottery. It was reported by several participants that safety netting would suffice, and parents could call back if anything changed/worsened. Overall, participants wrote that distance should not make a difference to decision making. | *“Transporting to hospital should not be based on distance if the child is well and no clinical concern. If they have worsened, they can call the ambulance back, distance should not make a difference to decision making”.*  *[PAR 7]* |
| Additional factors | ‘Inclusivity for all’  When asked about whether the tool should not be used in children with a developmental delay, it was clear from the free text comments that these are a difficult set of patients, but it would be discriminatory if the tool did not include these patients. Several comments suggest that the tool should be used in conjunction with parents/caregivers who know the patient well and what is normal for them. | *“This risks being discriminatory. I think various surrogates can be used as well”.*  *[PAR 36]* |
| Additional factors | ‘Newly qualified’  When asked if the tool should be available to all levels of paramedics including newly qualified paramedics, free comments were unanimous in that the tool should be used by all, particularly newly qualified paramedics. | *“I feel that a tool like this would be even more important to the newly qualified, unexperienced paramedic”.*  *[PAR 26]* |
| Additional factors | ‘Staying is not necessary’  When asked about whether paramedics should stay on scene for an hour to observe the child, participants in Round One initially suggested that paramedics would not have the time for this. The statement was modified for Round Two to consider that it may take less time off the road to observe for an hour, than to convey the child to hospital for observation. However, free text comments show that participants believe that this is not necessary. Additionally, it was commonly reported that by the time the paramedic had completed their examination and paperwork, an hour would have passed. This is coupled with the fact that most head injuries will not be attended to by a paramedic for over 30 minutes (unless red flags are reported). | *“I don’t agree that they should stay on scene for this long if they can safety net well. However, by the time the history, examination, treatment plan, paperwork and safety netting is undertaken then this normally takes this period of time so observation can be achieved then”.*  *[PAR 10]* |
| Additional factors | ‘Constructive feedback’  Paramedics do not often find out what happens to the patients they convey to hospital and therefore it is difficult to reflect on practice. The participants reported that feedback would be very helpful when considering head-injured children to improve decision making in the future. However, participants were clear that the feedback must be constructive and not critical. Some participants reported that there is a system in place in certain hospitals to provide feedback to paramedics. | *“Feedback is really important and will likely improve confidence in the future, especially non-conveyance if clinicians can identify that no clinical intervention is generally made, although this is likely to be more important in those just in case conveyances that occur, when there are no red flags present”.*  *[PAR 13]* |
| Format | ‘Paper is still needed’  Participants felt that the tool should be available both electronically and on paper. Most participants reported that flowcharts are usually easy to follow and that it is essential for this tool to be easy to follow, so that important information is not missed. | *“It should be available in both paper and electronic format to allow for those still using paper and also when there are electronic issues”*  *[PAR 11]* |
| Format | ‘Flow chart or App’  It was not clear whether participants preferred a flow chart or electronic progressive web application format. | *“It could work as a flow chart or as an exclusion risk (like the perc rule) in which if you say yes to any of the proposed sentences then you have a red flag”.*  *[PAR 29]*  *“A flow chart may not be possible, it would be complex and need to be well created”.*  *[PAR 6]* |
